# Evaluation of signal peptide prediction algorithms for identification of mycobacterial signal peptides using sequence data from proteomic methods

**DOI:** 10.1099/mic.0.025270-0

**Published:** 2009-07

**Authors:** Nils Anders Leversen, Gustavo A. de Souza, Hiwa Målen, Swati Prasad, Inge Jonassen, Harald G. Wiker

**Affiliations:** 1 Section of Microbiology and Immunology, The Gade Institute, University of Bergen, N-5021 Bergen, Norway; 2 Department of Informatics and Computational Biology Unit, BCCS, University of Bergen, N-5020 Bergen, Norway; 3 Department of Microbiology and Immunology, Haukeland University Hospital, N-5021 Bergen, Norway

## Abstract

Secreted proteins play an important part in the pathogenicity of *Mycobacterium tuberculosis*, and are the primary source of vaccine and diagnostic candidates. A majority of these proteins are exported via the signal peptidase I-dependent pathway, and have a signal peptide that is cleaved off during the secretion process. Sequence similarities within signal peptides have spurred the development of several algorithms for predicting their presence as well as the respective cleavage sites. For proteins exported via this pathway, algorithms exist for eukaryotes, and for Gram-negative and Gram-positive bacteria. However, the unique structure of the mycobacterial membrane raises the question of whether the existing algorithms are suitable for predicting signal peptides within mycobacterial proteins. In this work, we have evaluated the performance of nine signal peptide prediction algorithms on a positive validation set, consisting of 57 proteins with a verified signal peptide and cleavage site, and a negative set, consisting of 61 proteins that have an N-terminal sequence that confirms the annotated translational start site. We found the hidden Markov model of SignalP v3.0 to be the best-performing algorithm for predicting the presence of a signal peptide in mycobacterial proteins. It predicted no false positives or false negatives, and predicted a correct cleavage site for 45 of the 57 proteins in the positive set. Based on these results, we used the hidden Markov model of SignalP v3.0 to analyse the 10 available annotated proteomes of mycobacterial species, including annotations of *M. tuberculosis* H37Rv from the Wellcome Trust Sanger Institute and the J. Craig Venter Institute (JCVI). When excluding proteins with transmembrane regions among the proteins predicted to harbour a signal peptide, we found between 7.8 and 10.5 % of the proteins in the proteomes to be putative secreted proteins. Interestingly, we observed a consistent difference in the percentage of predicted proteins between the Sanger Institute and JCVI. We have determined the most valuable algorithm for predicting signal peptidase I-processed proteins of *M. tuberculosis*, and used this algorithm to estimate the number of mycobacterial proteins with the potential to be exported via this pathway.

## INTRODUCTION

The pathogen *Mycobacterium tuberculosis* is believed to be responsible for approximately 2 million deaths every year, the majority of which occur in areas with a large population density and poor health service infrastructure (WHO, 2007). The bacteria are typically transmitted via aerosol droplets generated from patients with an active form of the disease. Following exposure and inhalation of the bacilli, they cross the lung epithelium, and are subsequently taken up via specific receptor recognition by alveolar macrophages and by other immune cells in the lung interstitium. In this process, both membrane and secreted proteins play a central role in the host–pathogen interaction. Secreted proteins are used for nutrient uptake, adherence to host proteins and modulation of the host immune response, and are ultimately instrumental in allowing *M. tuberculosis* to target, and survive and proliferate inside immune cells such as macrophages and dendritic cells. Exposure to many of these proteins is evident from the immune response towards recognized secreted mycobacterial proteins (Målen *et al.*, 2008) and is considered to confer protection against subsequent infections with mycobacterial strains (Wiker *et al.*, 1986). Early observations demonstrated the importance of secreted proteins for the efficacy of the BCG vaccine (Heimbeck, 1948), and their significance is also reflected in the current vaccine phase II and III trial candidates, which consist of modified versions of a live BCG organism, or fusion proteins of extracellular proteins (Andersen, 2007). However, our knowledge of the proteins that make up the mycobacterial secretome does not quite match the importance that we attach to it, and is primarily based on the experimental identification of proteins produced and exported in axenic cultures in the laboratory.

The *Mycobacteriaceae* have several methods for releasing their proteins to the exterior, and lately there has been much focus on what has been suggested to be a type VII secretion system (for a review, see Abdallah *et al.*, 2007). However, the bulk of exported proteins are dependent on an N-terminal secretion signal and the activity of signal peptidase I. The signal peptide is normally made up of ∼ 30 aa, and is recognized by SecA, which transports the signal peptide-containing protein to a membrane-spanning complex that includes the proteins SecE and SecY. While the signal peptide is inserted into the membrane, the remainder of the protein is translocated across the membrane via the protein complex in an unfolded configuration. As the signal peptide is cleaved off, the mature protein can be released to the exterior of the cell. The N-terminal signal sequence starts with a positively charged region, and is followed by a hydrophobic region, and then a C-terminal region, which includes the cleavage site. The −3 and −1 positions relative to the mature protein appear to be of significance for the exact positioning of the cleavage event. It is the recognition of these shared features that has paved the way for development of algorithms to predict the presence of signal sequences and cleavage sites in proteins based on their N-terminal amino acid sequences. Many computational approaches exist to gain insights into the properties of proteins based on their sequences (Chou, 2002). The most specialized methods with regard to signal peptide prediction both predict the presence of a signal peptide sequence and suggest a probable cleavage site (von Heijne, 1986; Pugsley, 1993). The programs are based on either weight matrices or machine learning methods, and are trained on data acquired from Swiss-Prot and other resources that contain information about experimentally verified N-terminal sequences of mature secreted proteins. They are divided into two or more algorithms, each specialized to recognize signal peptides of certain groups of organisms, e.g. eukaryotes, Gram-negative bacteria and Gram-positive bacteria.

Until recently, relatively few secreted mycobacterial proteins with a known start of the mature sequence after cleavage by signal peptidase I were known. We have extended this set of proteins considerably (Målen *et al.*, 2007; de Souza *et al.*, 2008), and improved the dataset further using high-accuracy MS combined with a database that allows identification of N-terminal mature peptides starting between amino acids 15 and 45 of the annotated translational start site. Combined with 14 previously published proteins, we obtained a positive validation set of 57 secreted proteins. In addition, we gathered information about proteins that are not transported by the general secretory pathway. This negative validation set thus consisted of 23 new and 38 previously published proteins with observed N-terminal sequences coinciding with the predicted translational start site.

The accuracy of different signal peptide prediction algorithms has been compared on broad groups of micro-organisms, either using test sets extracted from protein databases, or by cross-validation methods that are commonly used during development of the algorithms (Menne *et al.*, 2000; Bendtsen *et al.*, 2004; Käll *et al.*, 2004; Zhang & Henzel, 2004; Shen & Chou, 2007; Chou & Shen, 2008).

The unique membrane of the *Mycobacteriaceae* made us question how these algorithms would perform on this group of organisms. Only a limited number of mycobacterial proteins have been included in the training sets for these algorithms, and it is important to note that the algorithms were developed with the broader purpose of identifying signal peptides of Gram-positive micro-organisms, and not to identify mycobacterial signal peptides as such. Against this background, and based on our extended experimental dataset of secreted mycobacterial proteins, we have investigated nine signal peptide prediction algorithms for their ability to predict the presence of a signal peptide and the respective cleavage sites. We found the hidden Markov model of SignalP v3.0 to perform best on our validation sets, and we used this algorithm to predict the repertoire of secreted proteins in mycobacterial species with available proteome annotations.

## METHODS

### Construction of validation sets.

A positive validation set was established, containing 57 proteins with a cleavage site following a putative signal peptide, based on previously published sequences (Harboe *et al.*, 1986; Nagai *et al.*, 1991; Sonnenberg & Belisle, 1997; Olsen *et al.*, 2000; Saleh & Belisle, 2000; Målen *et al.*, 2007; de Souza *et al.*, 2008) as well as novel data. In order to identify the signal peptidase cleavage sites of secreted proteins, we constructed a database for all annotated proteins of *M. tuberculosis* H37Rv. All N-terminal peptides created by cleavages between position 15 to 45 were added at the end of each protein and separated with the letter J, which does not code for an amino acid. The Mascot engine was programmed to treat the J as a tryptic site. The length of each added peptide was limited at the second tryptic site, thus allowing for one miscleavage. Using this database, we reanalysed our tandem mass spectrometric experimental data of *M. tuberculosis* H37Rv culture filtrates obtained on an LTQ-Orbitrap mass spectrometer (de Souza *et al.*, 2008). This culture filtrate is highly enriched for secreted proteins. We identified peptides that confirmed proteolytic cleavages for 40 proteins. For six of these proteins, none of the signal peptide prediction algorithms included in this study reported a signal peptide. We then analysed our total repertoire of peptides identified in *M. tuberculosis* to look for the occurrence of peptides at the N-terminal side of the identified proteolytic cleavage. In five of these six proteins, such peptides were identified, and based on a total evaluation we chose not to include any of these six proteins in our positive validation set. We also confirmed that no proteomics data identified peptides contained in the set of signal peptides predicted in the positive validation set.

We also created a negative set, containing proteins starting at the annotated translational start site (Muno *et al.*, 1981; De Bruyn *et al.*, 1987; Nagai *et al.*, 1991; Lee *et al.*, 1992; Harth *et al.*, 1994; Sørensen *et al.*, 1995; Menozzi *et al.*, 1996; Rosenkrands *et al.*, 2000; Målen *et al.*, 2007; de Souza *et al.*, 2008). The two sets are shown in Supplementary Table S1. The N-terminal sequences were taken from a combination of old and recently published experimentally verified N-terminal sequences, and we applied the following criteria for inclusion: N-terminal sequences determined by Edman degradation were included, and for sequences acquired by modern proteomic methods, only sequence data obtained with tandem MS were included. Of the latter, most sequences came from the study performed by de Souza and co-workers on culture filtrate proteins of *M. tuberculosis* H37Rv, which permitted us to extend the positive set from 37 to 53 proteins, and the negative set from 39 to 61 proteins (de Souza *et al.*, 2008).

### Protein sequence data.

The *M. tuberculosis* H37Rv laboratory strain protein sequences making up the proteome in this study were retrieved from the genome sequence published in 1998 (Cole *et al.*, 1998), with the updated annotation from 2002 (Camus *et al.*, 2002), both from the Wellcome Trust Sanger Institute. In addition, we used the J. Craig Venter Institute (JCVI) annotation, available at the JCVI Comprehensive Microbial Resource (http://cmr.jcvi.org/cgi-bin/CMR/GenomePage.cgi?org=ntmt02). From the Sanger Institute we also used the proteome annotations of *Mycobacterium bovis* subsp. *bovis* AF2122/97, *M. bovis* BCG Pasteur 1173P2, *Mycobacterium. leprae* and *Mycobacterium marinum*. JCVI sequencing and annotation was used for the strains *Mycobacterium avium* 104 (http://cmr.jcvi.org/cgi-bin/CMR/GenomePage.cgi?org=gma), *Mycobacterium smegmatis* MC2 (http://cmr.jcvi.org/cgi-bin/CMR/GenomePage.cgi?org=gms) and *M. tuberculosis* CDC1551. Sequences from *M. avium* subsp. *paratuberculosis* were taken from data published by Li *et al.* (2005), and sequences of *M. tuberculosis* H37Ra were made available by the Beijing Genomic Institute (http://gib.genes.nig.ac.jp/single/main.php?spid=Mtub_H37RA).

### Prediction algorithms.

The algorithms used in our analysis included SIGCLEAVE, PrediSi, SPEPLip, Signal-CF, Signal-3L, and the hidden Markov model and neural network method from versions 2 and 3 of SignalP. All are publicly accessible via the respective web interfaces, with the exception of Signal-CF, Signal-3L and SPEPLip, and allow the uploading of fasta files, making them appropriate for large dataset analyses. It has been found that mycobacterial signal peptides are most closely related to signal peptides of Gram-positive organisms (Wiker *et al.*, 2000), so this option was chosen for all programs, except for SIGCLEAVE, which only features choices for eukaryote and prokaryote versions.

SIGCLEAVE was previously part of the GCG Sequence Analysis package, but has been made freely available via EMBOSS since 1999. We used the web form hosted by the Pasteur Institute (http://bioweb2.pasteur.fr/) for our analyses. The program is based on the weight matrix method developed by von Heijne more than 20 years ago (von Heijne, 1986, 1987). The program was run with default settings using a minimum scoring weight value of 3.5, and the cleavage site with the highest score was assumed to be correct.

The PrediSi algorithm is available from the Institute for Microbiology, Technical University of Braunschweig, Germany (http://www.predisi.de/). It is based on a position weight matrix, where amino acid bias has been taken into account. Its training set was extracted from Swiss-Prot release 42.9.

The newly developed Signal-CF of Chou and Shen is available as a web server (http://chou.med.harvard.edu/bioinf/Signal-CF/ or http://www.csbio.sjtu.edu.cn/bioinf/Signal-CF) (Chou & Shen, 2007). Their dataset was extracted with a set of stringent conditions from release 50.7 of Swiss-Prot, to achieve what they describe as ‘high-quality benchmark datasets for eukaryotic, Gram-positive, and Gram-negative proteins’. It first determines whether the input sequence contains a signal peptide, and if so, employs a flexible scaled window in conjunction with a voting system to predict the cleavage site, with the intention of removing prediction bias. The Signal-3L algorithm (http://chou.med.harvard.edu/bioinf/Signal-3L/) represents a further development of Signal-CF, adding a third layer to increase prediction accuracy (Shen & Chou, 2007).

In 1997, Nielsen and co-workers published SignalP v1.0, which utilizes a machine learning approach based on neural networks for prediction of signal peptides and their cleavage sites (Nielsen *et al.*, 1997b). SignalP v2.0 was made available in 1999, and introduced an additional prediction algorithm, the hidden Markov model (Nielsen & Krogh, 1998). This version is still accessible, and can be found at the Centre for Biological Sequence Analysis, Technical University of Denmark (http://www.cbs.dtu.dk/services/SignalP-2.0/). For version 3 of SignalP (http://www.cbs.dtu.dk/services/SignalP/), the authors retrained the algorithms on a cleaned-up dataset, where previously inaccurately defined cleavage sites were removed. Although training on the improved dataset was the only change to the hidden Markov model, the neural network has seen an upgrade in its features and the introduction of a new score, D, for classification of signal peptides (Bendtsen *et al.*, 2004). We have used versions 2 and 3 in our studies. For all algorithms from SignalP, the submitted sequences were truncated at 70 amino acids from the N-terminal.

SPEPlip, created by Fariselli *et al.* (2003), uses the same neural network architecture as found in SignalP v2.0, but employs an updated training set. It searches for a putative signal peptide and suggests a cleavage site, and additionally searches for a PS00013 PROSITE pattern to determine whether the analysed sequence is a lipoprotein.

Prediction of transmembrane helices was performed using the TMHMM Server version 2.0, also found at the Centre for Biological Sequence Analysis, Technical University of Denmark (http://www.cbs.dtu.dk/services/TMHMM/) (Krogh *et al.*, 2001). Proteins predicted to have no transmembrane region, and proteins predicted to harbour a single transmembrane region within the first 60 amino acids, likely to represent the hydrophobic region of a signal peptide, were regarded as putative secreted proteins.

## RESULTS

### Performance of algorithms on validation sets

We used the positive and negative validation sets to determine how the algorithms performed in terms of finding the presence of a signal peptide within the protein sequences, and predicting the observed cleavage sites for proteins within the positive validation set. The results are presented in Table 1.

#### SignalP versions 2 and 3.

**Fig. 1. f1:**
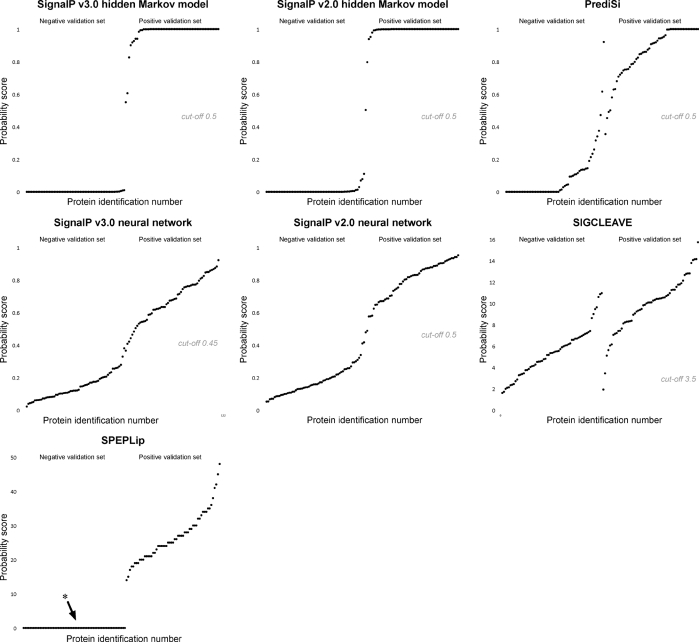
Scatter diagrams of signal peptide probability scores: the negative set is displayed on the left-hand half of the *x* axis, and the positive set on the right-hand half. The values from each set are sorted in increasing order by their acquired probability scores. Ideally, the scores should appear in separate ranges without overlap. The asterisk shows that the SPEPLip program does not output scores for proteins with a negative signal peptide prediction, and these proteins have therefore been given a minimum score (0), in order to plot them on the diagram.

The hidden Markov model of SignalP v3.0 showed the best overall performance at predicting signal peptides within mycobacterial proteins. All proteins within the positive set were predicted to be secreted, and all proteins in the negative set acquired a negative prediction. With a cleavage site prediction concordant with the observed site for 45 of the 57 proteins in the positive validation set, the hidden Markov model of SignalP v3.0 was also the best algorithm for predicting cleavage sites in our analyses, and this prediction was significantly improved compared to version 2 of the algorithm. Despite being outperformed by the latest version, SignalP v2.0 of the hidden Markov model was the second-best algorithm, with 39 of the observed cleavage sites being predicted. Version 3 of SignalP predicted the observed cleavage site of six proteins that had a different prediction in version 2 of the SignalP hidden Markov model, but there was also one example of a cleavage site correctly predicted by version 2 but incorrectly by version 3. Fig. 1 illustrates how proteins within the positive and negative validation sets were divided into two distinct groups based on their signal peptide probability scores. Version 3 had been trained with an improved training set leading to a lowered mean score in the negative set. However, we also saw examples of proteins in the positive set that had acquired a lower score in the most recent version. One protein in particular, Rv0999, acquired a new score of 0.551, bringing it very close to the cut-off of 0.5, from 0.797 in version 2 of the algorithm. We also saw an example of the opposite: the protein Rv0519c was given the score 0.503 in version 2, a score raised to 0.606 in version 3.

In contrast to the hidden Markov model of SignalP, the neural network algorithm actually saw a decline in performance from version 2 to version 3, where the most significant change was the four false negatives predicted by version 3, whereas there were none in the former version. There were nine discordant cleavage site predictions between SignalP v2.0 and v3.0. Of these, version 2 predicted five sites correctly, while version 3 predicted four cleavage sites concordant with the observed sites. Alltogether, version 3 predicted 29 of the 57 observed cleavage sites, while version 2 faired marginally better with 32 cleavage sites. Compared to the hidden Markov model, the distribution of scores outputted by the neural network program divided less markedly the negative and positive sets into two groups (Fig. 1). Interestingly, the groups were separated better in neural network version 2 than in version 3. The figure illustrates well how the stricter criteria used by the newest version have led to several instances in which the signal peptide probability score is reduced. Intriguingly, this seems to have had a greater impact on the scores of proteins within the positive set, leading to the inclusion of four false negatives with scores below the cut-off of 0.45. SignalP provides a separate score for prediction of cleavage sites, which in version 3 is represented by the Y-max score for the neural network and C-max for the hidden Markov model. The score is given regardless of whether the final prediction is negative or positive, and in our analysis we decided to include this score from proteins in the positive validation set that acquired a negative signal peptide prediction.

The neural network-specific Y-max score is derived from a combination of two separate scores: the C-score, reflecting the probability of a position being at the cleavage site; and the S-score, given to positions towards the end of the signal peptide. Furthermore, the signal peptide probability score for the neural network model, D, is an average of the Y-max and S-mean scores. In contrast, the cleavage site score, C-max, from the hidden Markov model appears to be independent of the signal peptide probability score, and varies greatly within the positive validation set. Incorporating signal peptide information in the cleavage site score, Y-max, was done to ensure that the cleavage site corresponded to the preceding signal peptide. In our set, we observed four instances where this information clearly contributed to improved predictions, by ignoring an alternative cleavage site with a higher cleavage site score, but whose position did not correspond to the trailing end of the signal peptide. However, we also observed five instances where a higher or identical C-score corresponded to the experimentally observed cleavage site, but when the signal peptide score was incorporated into Y-max, the resulting cleavage site prediction was different.

It was unexpected to see the hidden Markov model perform better than the neural network algorithm on our validation sets, as the opposite trend has been described elsewhere (Menne *et al.*, 2000; Bendtsen *et al.*, 2004; Käll *et al.*, 2004; Zhang & Henzel, 2004). The difference could be related to the length of the signal peptides, which were slightly longer than a typical Gram-positive signal peptide (von Heijne & Abrahmsen, 1989). The hidden Markov model predicted more correct cleavage sites for signal peptides that were shorter, and longer, than the average length. However, the neural network algorithm performed particularly poorly on proteins with long signal peptides. Comparison of two independent annotations of the *M. tuberculosis* H37Rv genome showed that there are considerable problems related to correct protein start site predictions (de Souza *et al.*, 2008). This has an impact on signal peptide length, which in turn favours an algorithm that is less prone to errors based on variation in the length of the signal peptide.

#### Signal-3L and Signal-CF.

In terms of cleavage site prediction, Signal-3L and Signal-CF were ‘runners up’ behind versions 2 and 3 of the hidden Markov model of SignalP, with 39 and 36 correct predictions of the observed sites, respectively. Although neither algorithm predicted false negatives, both predicted the same nine false positives from the negative validation set. Overall, we saw an improved performance for Signal-3L over Signal-CF. Neither of the two algorithms outputs a prediction score.

#### PrediSi.

With 32 predicted cleavage sites concordant with the observed number, PrediSi performed marginally poorer than Signal-CF, but in turn only predicted two false positives. However, it did also predict three false negatives from the positive set. To a higher degree than the neural network of SignalP, PrediSi did polarize the scores from the two validation sets (see Fig. 1). A majority of the negative, and approximately half the positive, were awarded a minimum score (0) and a maximum score (1), respectively.

#### SPEPLip.

SPEPLip performed very well in terms of prediction of the presence of a signal peptide, with only one false negative and no false positives. However, the cleavage site prediction algorithm performed relatively poorly, with only 21 of the cleavage sites correctly predicted. The program does not output scores for proteins with a negative signal peptide prediction, nor does the documentation reveal information about the cut-off that has been used to discriminate between the two groups. However, the distribution of the positive scores indicates that the scores are not clearly separated, unlike the scores for the hidden Markov model of SignalP (Fig. 1).

#### SIGCLEAVE.

The SIGCLEAVE algorithm performed poorly on the mycobacterial validation sets. It predicted only 21 of the 57 observed cleavage sites, but perhaps the most noteworthy observation was the 47 false-positive predictions in the negative set. Fig. 1 outlines the distribution of scores in the negative and positive validation sets, and a significant overlap of scores can be observed. By increasing the cut-off to 7.5, the algorithm would fare better, with only seven false positives. On the other hand, this would also result in 11 false-negative predictions.

### Prediction of secreted proteins in mycobacterial proteomes

Taken together, our results showed that the Gram-positive bacteria-specific hidden Markov model algorithm of SignalP v3.0 is currently the best program for predicting the presence of a signal peptide in mycobacterial proteins.

We therefore used this algorithm for a proteome-wide analysis of 11 available proteome annotations, including the two annotations of *M. tuberculosis* H37Rv. We further used TMHMM v2.0 to identify proteins containing transmembrane regions, which would suggest that they are associated with the membrane rather than being secreted. The results are presented in Table 2. Combining the two algorithms, between 7.78 and 10.47 % of the proteins were predicted to be secreted. The highest percentage of secreted proteins was seen in *M. leprae*, although this organism could be considered a special case, due to the high number of pseudogenes. Interestingly, for this proteome, only 0.37 % of the proteins were both predicted to be secreted and to contain transmembrane regions downstream of the signal peptide.

When analysing signal peptide prediction in mycobacterial proteomes, we observed a consistent difference in the percentage of predicted proteins in the annotations between the Sanger Centre and the JCVI. This is best exemplified by the annotations for the *M. tuberculosis* H37Rv strain, where 9.62 % were predicted as secreted in the Sanger annotation, while only 7.89 % were predicted in the JCVI annotation. To better understand the reason for this discrepancy, we divided the proteins with positive predictions from the two annotations into three groups: (1) proteins with identical sequences in both annotations; (2) proteins that are unique to the specific annotation; and (3) proteins that share the same stop codon, but that have been annotated with a different start site. There were 227 proteins predicted to harbour an N-terminal signal peptide, and that were shared between the annotations. The Sanger annotation had 90 unique proteins that obtained a positive prediction, while the JCVI annotation had 100. The most marked difference was observed for corresponding proteins with different start codons between the annotations: from the Sanger annotation, a total of 204 from this group of proteins were predicted to have a signal peptide, while only 133 proteins from the JCVI annotation received a positive prediction. Although this observation in itself might not be indicative of the quality of the two annotations, the N-terminal region is a conserved feature of a significant number of proteins. Thus, the finding of a higher number of positively predicted proteins within annotations from the Sanger Institute for the *M. tuberculosis* H37Rv strain could indicate that this annotation has a higher number of correct annotations for translational start sites. This is supported by the recent findings of de Souza and co-workers, who used high-quality MS to verify experimentally several peptides unique to the Sanger annotation, while only finding one peptide unique to the JCVI annotation (de Souza *et al.*, 2008). This is a further reminder that the output of the signal peptide prediction algorithms depends not only on the program itself but also on the quality of the input protein sequences.

## DISCUSSION

We have shown that for the proteins in our validation set, the existing signal peptide-prediction algorithms vary greatly in their ability to predict N-terminal signal peptides and the respective cleavage sites for mycobacterial proteins. Our observations lead us to conclude that the hidden Markov model of SignalP v3.0 is the best predictor of mycobacterial signal peptides. This algorithm was tested against other available programs that we thought to be relevant and that can be freely accessed via their web interfaces. We are aware of at least one algorithm available under a commercial licence, SPScan, that we have not had the opportunity to test using our validation sets. However, a comparison of signal peptide-prediction algorithms published by Menne *et al.* (2000) showed an overall poorer performance of this program than that of the SignalP v2.0 neural network method and the hidden Markov model, when tested on a large trans-species validation set. It did provide the lowest number of false positives in the negative set, but this result was marred by its producing the greatest number of false negatives in the positive set. They also tested SignalP v1.1 and SIGCLEAVE in their analysis. The validation sets used in their article grouped together sequences from eukaryotes and prokaryotes, and the cleavage site algorithms performed markedly better than in our study of mycobacterial proteins. Within their positive validation set, correct cleavage sites were observed for 82.6 % of the proteins using the SignalP v2.0 hidden Markov model, and 84.6 % of the proteins using the SignalP v2.0 neural network approach, which may be compared to the values observed in our positive validation set, which were 68.4 and 56.1 %, respectively.

The creators of the SignalP algorithm have observed that algorithms trained on sequences from species within a group of organisms, e.g. eukaryotes, Gram-negatives or Gram-positives, perform better than algorithms trained on a single organism (Nielsen *et al.*, 1997a). The family *Mycobacteriaceae* might be in an exceptional position, belonging to the group of Gram-positive species, but with a complex outer membrane that has yet to be fully elucidated (Zuber *et al.*, 2008). The question therefore is whether the signal peptides of mycobacteria have features that distinguish them from their Gram-positive counterparts, and if so, whether there is a case for optimizing signal peptide algorithms by training them on more appropriate protein sets. Against this, we found the SignalP v3.0 hidden Markov model to perform reasonably well, and having been trained on the same protein sequences, it is clearly the algorithm itself, and not the training set, that differentiates the hidden Markov model from the neural network method of SignalP.

The SignalP v3.0 hidden Markov model missed the observed cleavage sites in the positive validation set in 12 cases. In four of these, the observed cleavage site was located upstream of the predicted cleavage site. In these cases the predicted cleavage sites are likely to be incorrect. In the other seven cases, the observed cleavage sites were located downstream of the predicted cleavage site. In such cases it is more difficult to rule out the predicted cleavage site, as the observed cleavages could be the result of secondary processing by another peptidase. In many cases the SignalP algorithm also predicted alternative cleavage sites, in addition to the suggested cleavage site. We found that of the 12 proteins with an erroneous cleavage site prediction by version 3 of the hidden Markov model, seven actually had registered a score for the observed cleavage site. We also saw seven examples of the observed cleavage site in close proximity (≤3 aa in either direction) to the predicted cleavage site. In fact, we have made experimental observations to show the occurrence of two cleavage sites in the same protein, Rv2253 (de Souza *et al.*, 2008). These sites are only one position apart, and both are predicted by the hidden Markov model of SignalP v3.0. It is possible that certain sequences in close proximity to the cleavage site allow for a more liberal positioning of the signal peptide, which in turn is a source of versions of the mature protein that differ in length by a small number of amino acids. It is not known whether the small variation in protein length has any biological significance.

We chose to include all available secreted proteins with observed N-terminal mature sequences in the positive validation. This included nine proteins that also were predicted to be lipoproteins with a correctly positioned PS13 lipoprotein consensus motif. However, LipoP, the lipoprotein prediction algorithm for Gram-negative bacteria from the Centre for Biological Sequence Analysis, Technical University of Denmark (Juncker *et al.*, 2003), defined six of these proteins as secreted via the general secretory pathway. Furthermore, the cleavage site of one of the three proteins predicted to be a lipoprotein was in fact correctly predicted by all the tested signal peptide prediction algorithms, with the exception of the SignalP v2.0 neural network method and SIGCLEAVE.

An advantage of the SignalP algorithm is that the complete output is available regardless of the prediction outcome, and it allows for a more rigorous analysis of proteins with ambiguous cleavage sites. We would certainly encourage developers of signal peptide prediction algorithms to be forthcoming with any quantitative information that is being used to make the final prediction, as it allows for in-depth analysis using validation sets, and might even lead to a better understanding of the cleavage and secretion event itself.

Compared to other studies that have validated signal peptide-prediction algorithms, our positive validation set may seem small. These studies have included proteins from a range of different genera. We have studied a relatively large number of secreted proteins with verified cleavage sites from the same organism, and we believe that the positive validation set is representative not only for secreted proteins of *M. tuberculosis* but also for mycobacteria in general.

The hidden Markov model of SignalP v3.0 showed the best overall performance for the mycobacterial protein dataset constructed in this paper. However, for the signal peptides in other datasets and of other organisms, a complementary combination with other powerful predictors such as Signal-3L and Signal-CF is needed, as indicated by Table 3 of Shen & Chou (2007). One may use the strategy of running several signal peptide-prediction algorithms to characterize a protein, and if many algorithms predict a signal peptide, one will be more confident about the result.

The secretory pathway represents quantitatively the most important protein secretion system, and it is essential to consider it when evaluating proteins that are exposed to, and interact with, proteins of the host. Furthermore, as these proteins are cleaved as part of the secretion process, knowing the exact sequences of the mature proteins is important for construction of recombinant proteins for evaluation of immunogenic properties, or when incorporating them into vaccines based on recombinant proteins. Although the hidden Markov model of SignalP v3.0 also performed best for cleavage site prediction, a cleavage site accuracy of 78.9 % shows that there is potential for improvement. Perhaps the unique structure of the mycobacterial cell wall is reflected in the signal peptide sequence of secreted mycobacterial proteins, and as such will require a more specialized prediction algorithm. On the other hand, further development of an algorithm for Gram-positive bacteria might also give better prediction of mycobacterial proteins.

## Figures and Tables

**Table 1. t1:** Signal peptide prediction by various algorithms on a positive and negative validation set

**Algorithm**	**Signal peptide prediction**		**Cleavage site prediction; identified sites in positive set (*n*=57)**
	**Positive predictions in positive set (*n*=57)**	**Negative predictions in negative set (*n*=61)**	
SignalP v3.0 hidden Markov model	57 (100.0 %)	61 (100.0 %)	45 (78.9 %)
SignalP v2.0 hidden Markov model	57 (100.0 %)	61 (100.0 %)	39 (68.4 %)
Signal-3L	57 (100.0 %)	52 (85.2 %)	39 (68.4 %)
Signal-CF	57 (100.0 %)	52 (85.2 %)	36 (63.2 %)
PrediSi	54 (94.7 %)	59 (96.7 %)	32 (56.1 %)
SignalP v2.0 neural network	57 (100.0 %)	61 (100.0 %)	32 (56.1 %)
SignalP v3.0 neural network	53 (93.0 %)	61 (100.0 %)	29 (50.9 %)
SPEPLip	56 (98.2 %)	61 (100.0 %)	21 (36.8 %)
SIGCLEAVE	55 (96.5 %)	14 (33.0 %)	21 (36.8 %)

**Table 2. t2:** Signal peptide prediction by SignalP v3.0 hidden Markov model for various mycobacterial proteome annotations

**Organisms**	**Total number of proteins**	**Predicted signal peptides (percentage of total)**	**Predicted signal peptides, no transmembrane region (percentage of total)**
*M. tuberculosis* H37Rv (Sanger)	3991	519 (13.0 %)	384 (9.6 %)
*M. tuberculosis* H37Rv (JCVI)	4219	464 (11.0 %)	333 (7.9 %)
*M. tuberculosis* H37Ra (Beijing)	3991	526 (13.2 %)	386 (9.7 %)
*M. tuberculosis* CDC1551 (JCVI)	4189	497 (11.9 %)	363 (8.7 %)
*M. bovis* subsp. *bovis* AF2122/97 (Sanger)	3920	515 (13.1 %)	379 (9.7 %)
*M. bovis* BCG Pasteur 1173P2 (Sanger)	3891	509 (13.1 %)	367 (9.4 %)
*M. avium* 104 (JCVI)	5245	581 (11.1 %)	413 (7.9 %)
*M. avium* subsp. *paratuberculosis* (University of Minnesota)	4350	532 (12.2 %)	378 (8.7 %)
*M. marinum* (Sanger)	5462	729 (13.3 %)	542 (9.9 %)
*M. leprae* (Sanger)	1605	174 (10.8 %)	168 (10.5 %)
*M. smegmatis* mc^2^ (JCVI)	6880	807 (11.7 %)	535 (7.8 %)

## Abbreviations

- JCVI, J. Craig Venter Institute
